# Technology-Enhanced Atmospheric Moistening (TEAM) for More Precipitation: A Perspective

**DOI:** 10.1021/acs.est.5c06428

**Published:** 2025-10-03

**Authors:** Sarah N. Warnau, Jolanda J. E. Theeuwen, Gholamabbas Sadeghi, Imme Benedict, Bert H. V. M. Hamelers, Chiel C. van Heerwaarden

**Affiliations:** † 361182 Wetsus, Leeuwarden 8900 CC, The Netherlands; ‡ Meteorology and Air Quality Group, 4508 Wageningen University & Research, Wageningen 6700 AA, The Netherlands; § Copernicus Institute, Utrecht University, Utrecht 3584 CS, The Netherlands; ∥ Environmental Technology Group, Wageningen University & Research, Wageningen 6700 AA, The Netherlands

**Keywords:** water scarcity, weather modification, precipitation enhancement, freshwater production, evaporation technology, atmospheric moisture recycling

## Abstract

Large-scale freshwater production solutions can alleviate freshwater scarcity and support ecosystem restoration. In this perspective, we propose Technology-Enhanced Atmospheric Moistening (TEAM) to increase regional precipitation, such as rain and snow, and boost large-scale freshwater availability. By enhancing moisture in the atmosphere, TEAM reduces the amount of atmospheric lifting needed for cloud formation. TEAM can be implemented through spray or solar evaporation technologies, each with their own challenges and opportunities. Using atmospheric moisture flows between evaporation sources and precipitation sinks, we estimate where coastal evaporation from seven source locations may result in precipitation. This is a starting point for the identification of areas with high and low TEAM precipitation enhancement potential. Then, we discuss the position of TEAM in the larger field of technologies for enhancing freshwater supply where we compare TEAM with desalination through reverse osmosis, cloud seeding, and forestation for precipitation enhancement. Further research should focus on (1) quantifying the potential for precipitation generation by enhanced atmospheric moistening, (2) developing technologies that can supply this moisture to the atmosphere, and (3) interdisciplinary regional design. With further research and development, we believe that TEAM can contribute to large-scale freshwater generation.

## Graphical abstract

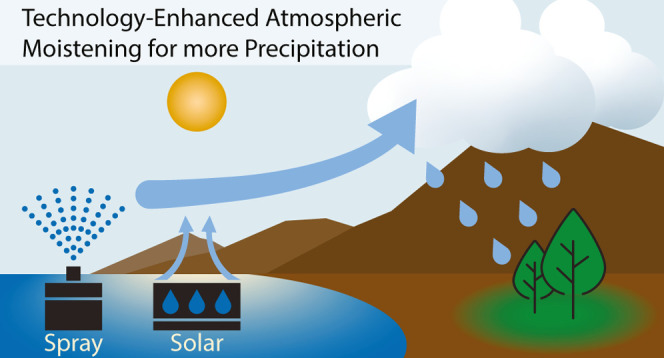

## Introduction

To deal with freshwater scarcity in dryland regions, we introduce a thus far unexplored technological option for large-scale freshwater generation: Technology-Enhanced Atmospheric Moistening (TEAM) for more precipitation. The approach entails the use of low-quality water, such as seawater, to technologically increase atmospheric moisture and thereby create high-quality freshwater in the form of precipitation such as rain or snow. The prospect is a technological system that can enhance freshwater availability sustainably on a large-scale in coastal dryland areas. The idea is supported by recent observational evidence suggesting that enhanced evaporation triggers additional rainfall 10–50 km downwind of extensively irrigated areas, with the strongest signal detected in arid regions.^1^

Freshwater scarcity is a pressing global issue driven by land use change, climate change, and an increasing demand due to population and economic growth.^2^ Terrestrial ecosystems such as forests play a crucial role in the local water cycle as they support infiltration and, under the right conditions, support local precipitation through evapotranspiration.^3,4^ Conversely, drylands tend to self-expand through similar land-atmosphere feedbacks.^5^ Feedback loops between environmental processes and socio-economic dynamics can drive regional water systems toward a collapsed state of anthropogenic drought.^6,7^ To improve overall freshwater security and restore degraded and moisture depleted ecosystems, additional sources are needed.

To meet increasing demand, a range of technologies has been developed to enhance freshwater availability. Cities and industries are progressively relying on desalination processes like reverse osmosis and electrodialysis to produce potable water from seawater.^8^ Other approaches, such as cloud seeding, attempt to enhance precipitation by modifying microphysical atmospheric processes,^9^ while strategic forestation can influence regional rainfall patterns by increasing evapotranspiration over large areas.^10^

Here, we propose and investigate TEAM as an alternative: by bringing more moisture into the atmosphere, eventually more precipitation will be produced. Using either direct solar radiation or atmospheric heat for the evaporation of seawater limits the amount of electrical energy needed for the operation of such an evaporation system, making it an interesting option for large-scale applications. In this paper, large-scale enhancement of freshwater by induced precipitation is considered over a substantial region, such as a country or a river basin.

This perspective delineates how the TEAM approach could be used to increase precipitation. First, we discuss the meteorological potential for precipitation enhancement. Then, we discuss the technological potential to moisten the atmosphere, where we discuss two methods of seawater evaporation: spraying and interfacial solar evaporation. Next, we make a first approximation of where the enhanced atmospheric moisture is expected to precipitate out using a data set of atmospheric moisture flows. After, we discuss the positioning of TEAM in the broader field of technologies for freshwater supply enhancement. We conclude with an outlook on further research.

## The Meteorological Potential for Precipitation Enhancement

For evaporated water to return to the ground as precipitation, the air that contains the water vapor needs to be lifted in order for condensation to occur. Since the atmospheric pressure decreases with height, the adiabatic lifting of air will cause expansion and thus cooling. At a certain height, which is called the lifting condensation level, the air has cooled to its dewpoint, i.e. the air is now saturated with water vapor. Lifting the air further will cause part of the water vapor to condense, leading to cloud formation. This condensation typically occurs on condensation nuclei, which can be aerosols such as dust and salt. For precipitation to form, cloud droplets (radius ∼ 10 μm) need to develop into rain droplets (radius ∼ 2000 μm).^9^ Moistening of air from the surface will increase the dew point temperature of the air and thus increase the chance that when air is lifted, the lifting condensation level is reached (Figure 1).

**Figure 1:**
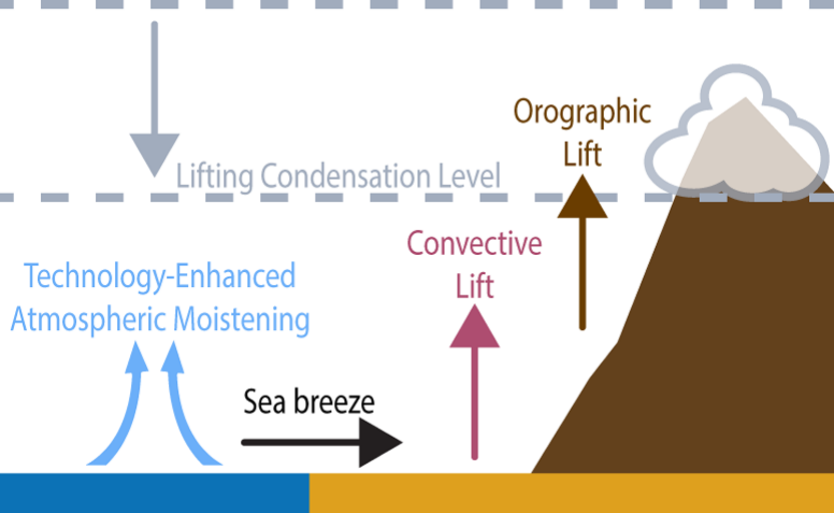
Conceptual illustration of Technology-Enhanced Atmospheric Moistening over sea and the atmospheric processes that promote subsequent cloud formation over land. The lifting condensation level lowers when air at the surface is moistened, increasing the chance that lifted air will reach this level and clouds will form. Lifting mechanisms are convection and orographic lift. Transport of air from sea to land can be provided by a sea breeze.

A region’s atmospheric and geographic conditions are expected to determine whether implementation of TEAM can successfully enhance regional precipitation. While there are many situations where TEAM may not increase precipitation locally, here we focus on conditions where it has the highest likelihood of success. First, there needs to be wind from the sea to the land, for example provided by a sea breeze, to transport the enhanced atmospheric moisture land-inward. A sea breeze is formed when during the day, the land heats up faster than the sea due to its lower heat capacity than water. The warm air over the land rises, creating a low-pressure area. To maintain mass balance, cooler, denser air from over the sea moves inland.^11^ Second, local atmospheric lifting mechanisms need to be present. The lifting of air can be caused by synoptic (large-scale) weather systems, orography, and convection. Since we are interested in a localized and relatively controlled effect we focus on orographic lift and convection.

The best time to implement TEAM is during the day in summer. A sea breeze is most likely to develop during the day, and also in the summer period, as the temperature gradient between land and sea is large.^11^ Additionally, in summer convection is strongest.^9^ Furthermore, the relative impact of enhanced precipitation is also largest in summer, when regions are at their driest. An example of a location where a decrease in summer precipitation has been linked to a lack of local evaporation is the Spanish Mediterranean coast, where a wind system develops during the day that combines a sea breeze and up-slope wind, and the lack of evaporation is linked to deforestation and urbanization.^12^

Quantifying how much of local evaporation will return as precipitation in a certain area depends on an interplay of processes on different scales: from forced orographic lift to cloud (thermo-)­dynamics and cloud microphysics. Furthermore, the stability of the atmosphere is key in determining if air from the surface can convectively be lifted.^9^ While the interactions between the land surface and the atmosphere and specifically between soil moisture and precipitation have been studied extensively,^13–18^ studies into the quantification of evaporation–precipitation relationships are limited. Thus, tools to quantify the effect of TEAM on regional precipitation will need to be developed.

Now that we have discussed the meteorological reasoning for us to believe that there is potential for precipitation enhancement using TEAM, in the next section we look into the question how to moisten the atmosphere using technology.

## Meeting the Atmospheric Moistening Demand through Technology

### Technology-Enhanced Moistening of the Atmospheric Boundary Layer

To meet the atmospheric demand for additional moisture to increase precipitation in a certain region, a technology needs to be developed. Lab experiments can show the ideal evaporation rate that a certain technology can output. However, the actual evaporation rate will depend on interactions of the technology with the atmosphere, specifically with the atmospheric boundary layer (ABL). The ABL is the lowest part of the atmosphere (∼100–3000 m) that is directly affected by the earth’s surface and reacts to surface forcings on time scales up to 1 h.^19^ ABL properties and processes relevant for the actual amount of atmospheric moistening are turbulent mixing, temperature, humidity, and ABL depth and growth. Feedbacks between the ABL and the technology will determine the effective moistening downwind. Here, we consider two technology types that can potentially be used for a TEAM application. We discuss the ideal performance and how feedbacks in the ABL are expected to impact the actual performance with large-scale implementation.

### Spray Evaporation

A straightforward way of enhancing the atmospheric moisture content is by spraying water directly into the air. Spray evaporation is widely used in sectors like construction, food, agriculture, power, and automotive,^20^ and as small-scale outdoor cooling applications in the built environment.^21^ Here, we want to explore the possibility of using large-scale spraying of seawater to increase the humidity of the atmosphere (Figure 2A).

**Figure 2:**
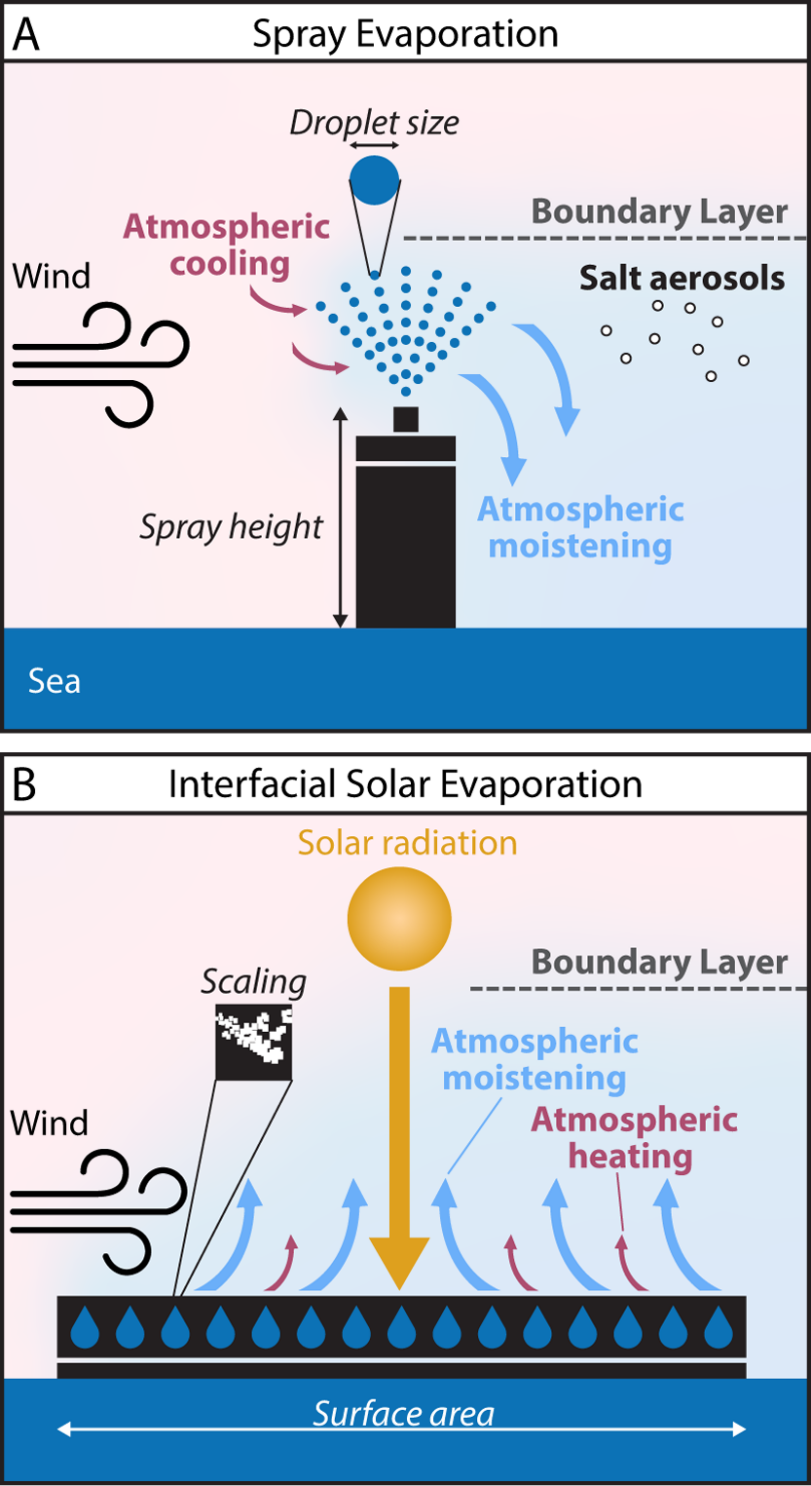
Spray (A) and solar (B) evaporation technologies, implemented on a sea surface. Italic text indicates technological challenges. Bold text indicates meteorological conditions and processes that impact the technology performance in the field and the downwind effective moistening.

A spray is created by pumping water through a nozzle at a certain height, creating water droplets in the air that fall back to the sea surface. While the water droplets are in the air, they capture heat from the surrounding air and use this energy for evaporation, thereby cooling and moistening the air, increasing the relative humidity, and lowering the lifting condensation level. If the air is cooled and moistened to saturation, the air will have obtained its wet bulb temperature. The adiabatic wet bulb temperature and resulting atmospheric moisture can be calculated or obtained from a psychometric chart if the atmospheric pressure and initial temperature and humidity are known. Thus, the maximum potential of atmospheric moistening from spraying is determined by the initial temperature and humidity of the air.

The technical challenge lies in optimizing the spray height and droplet size to get a maximum moistening effect while limiting the operational costs. On the one hand, droplets that return to the sea surface represent an energy loss. On the other hand, droplets should not evaporate fully before reaching the sea surface, as to return the dissolved salt to the sea and prevent the formation of sea salt aerosols, to limit the risk of soil salinization downwind. The role that sea salt aerosols can play in cloud processes as cloud condensation nuclei is outside the scope of this perspective. The evaporation speed and drift of droplets is further impacted by the wind speed, which prolongs the atmospheric suspension of the droplets.^22^ Furthermore, there are ABL feedbacks to be considered. Large-scale spray evaporation could cause the local ABL to become less turbulent and more stable because of the cooling effect, as is observed over irrigated fields,^23^ which could limit convection.

### Solar Evaporation

The second type of technology we consider is interfacial solar evaporation (Figure 2B). The main practical application is seawater desalination where the produced water vapor is collected on a condenser.^24,25^ Other potential applications include wastewater management, sterilization, and power generation.^25^ Solar evaporation happens when the radiation from the sun increases the water temperature enhancing surface evaporation. The natural evaporation rate of the ocean is largely dependent on the sea surface temperature and the wind speed.^26^ The global mean, annual mean evaporation rate of the ocean is ∼3 kg m^–2^ day^–1^ while regionally it can range from ∼1 to 7 kg m^–2^ day^–1^.^27^ To technologically increase the evaporation rate, heat localization can be implemented to maintain the heat at a confined layer at the top of the water.^28^ Also, the reflectivity of the surface can be lowered, to capture as much solar radiation as possible. Reported efficiencies (ratio of latent heat flux of evaporation to solar radiation flux) of heat localized solar evaporation systems of different materials and shapes are typically ≥78% for lab conditions under 1000 W m^–2^ solar intensity,^29^ which corresponds to ≥1.12 kg m^–2^ hour^–1^.

The main technological challenges for solar evaporation systems are salt deposition on the evaporator surface,^30^ scaling up the systems while maintaining high evaporation rates,^28^ and how to limit the area footprint of a large solar evaporation system. To lower the area footprint, 3D systems are designed, increasing the evaporative area per surface area. Very high (up to 34 kg m^–2^ hour^–1^) evaporation rates are reported for a 3D graphene oxide stalk which was well ventilated (windspeed = 3.5 m s^–1^).^31^ To reach this high evaporation rate, heat from the environment plays a crucial role,^31^ which makes this type of solar evaporation system more similar to a spray system since it cools the environment. For large-scale systems, it is expected that the downwind efficiency of the evaporator will decline because of upwind moistening.^32^ This decline will depend on local ABL mixing and depth.

## From Evaporation to Precipitation: Where Will It Rain Out?

Now that we have described two technological options for enhancing atmospheric moisture, we continue with a first estimate of how much and where to expect enhanced precipitation with an implementation of TEAM. We use an atmospheric moisture connections data set, which describes the moisture flows between evaporation sources and precipitation sinks, thus giving an estimate of the potential region of impact of the technology. The region where evaporation from one source location precipitates out is called the evaporation footprint.^33^ Moisture connection data obtained from ERA5 reanalysis data using the Lagrangian atmospheric moisture tracking model UTrack^33^ is freely available as multiyear (2008–2017) monthly means at a spatial resolution of 0.5° × 0.5°.^34^ From this data set, we examine the evaporation footprints of seven source locations spread over the globe, four in the Northern Hemisphere and three in the Southern Hemisphere. The locations were selected to ensure global coverage and represent a diverse set of coastal drylands. Coastal drylands have high seawater availability, and high potential to benefit from enhanced precipitation, since in arid and semiarid regions a lack of soil moisture is the leading limitation for ecosystem restoration.^4^ The selected evaporation sources are located on the sea and close to the coast of the Los Angeles Basin in the United States, Valencia in Spain, Aden in Yemen, Bohai Wan in China, Coquimbo in Chile, Cape Town in South Africa, and Perth in Australia (Figure 3). Through the analysis of the shapes and sizes of the evaporation footprints and the relations thereof with geographical or meteorological features, we can assess whether natural conditions facilitate or hinder the regional precipitation of added moisture.

**Figure 3:**
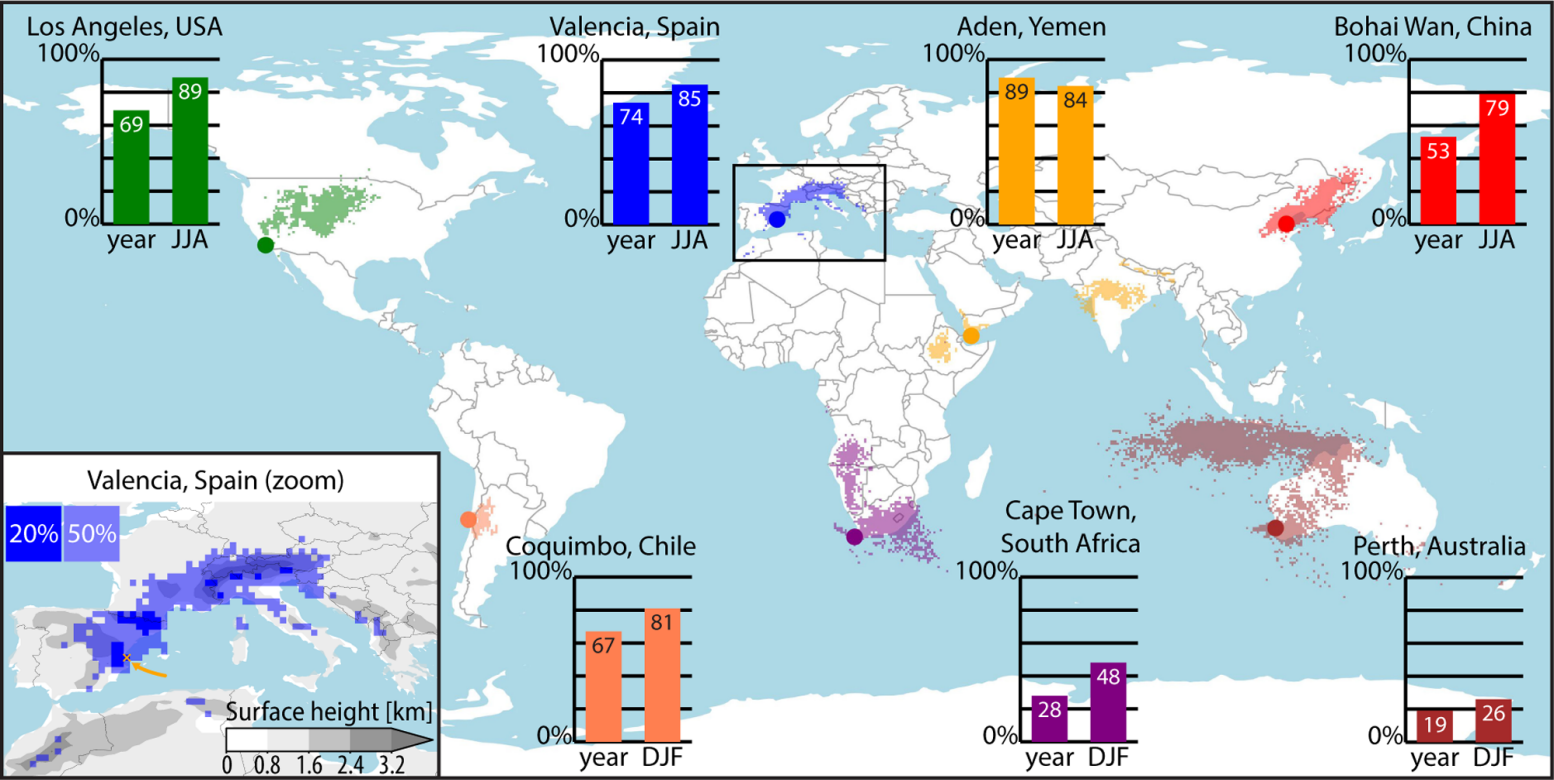
World map of evaporation footprints of seven source locations near coastal drylands around the world. Bar graphs show yearly mean (left bar) and summer mean (right bar) land precipitation fraction (%): the fraction of evaporation per source location that precipitates out on land. The means are weighed by the total monthly evaporation. For locations in the Northern Hemisphere, the months June–August (JJA) are chosen as summer, and for the Southern Hemisphere December–February (DJF). The global map shows the 50% evaporation footprints of the source locations (dots) during the summer. The inset shows a zoom of the Valencia footprint where the 20% footprint is added in dark blue and the evaporation source is indicated with a yellow cross and arrow. Filled gray contours show the land surface height in kilometers.

First, we determine for every source location the percentage of precipitation that falls over land, as an average over the year and for the summer period only (June–August in the Northern Hemisphere and December–February in the Southern Hemisphere). We observe that the fraction of the evaporation from the source locations that precipitates over land is generally high (Figure 3, bar graphs), especially during the summer with a median of 81% averaged over all locations. Only for the locations near Cape Town and Perth, the land precipitation fraction is less than 50%. Furthermore, the location near Aden is the only location where the land precipitation fraction is not higher in summer than in the yearly mean.

Second, we determine the 50% evaporation footprints of the source locations during the summer. These evaporation footprints show the smallest amount of 0.5° × 0.5° grid cells where 50% of the evaporation source has precipitated out, hence, these grid cells receive the largest fraction of evaporation from the source location (Figure 3, global map). The sizes and spatial patterns of the footprints are vastly different between the different source locations. For example, the Coquimbo footprint is relatively small and contained near the source location, while the Aden footprint is spread over three countries: Yemen, Ethiopia, and India. For certain source locations, e.g. near Aden, Cape Town, and Perth, the shape and extend of the footprint seem to be controlled by large-scale atmospheric circulation such as westerlies or trade winds. For source locations with a smaller and more localized footprint, the local geography seems more important. Zooming in on the Valencia footprint, we observe that the bulk of the 20% evaporation footprint is found over the mountain range close to the coast, and over the Pyrenees mountains (Figure 3, inset). This suggests that the presence of mountains close to the evaporation source contributes to the localization of the precipitation effect, as has been found in previous analysis of this data set as well.^35^ Furthermore, the footprints show that the regional efficiency in the conversion of evaporation to precipitation not only depends on the source location but also on the spatial extent of the targeted area, as larger regions tend to capture a larger fraction of the source’s moisture.

With these footprints, we have obtained an initial indication of source locations where the probability of regional-scale precipitation enhancement with TEAM is high (e.g., Coquimbo, Valencia, and Bohai Wan) or low (e.g., Perth). In addition, we find that a large fraction of the evaporated moisture from potential source regions precipitates over land. Moisture connections thus provide a first approximation of regions potentially suitable for TEAM and of its possible range of impact. This can also indicate the source areas (e.g., Aden) where the chance is high that TEAM will increase precipitation in already wet regions. Further research with moisture tracking models could explore the sensitivity of the evaporation footprint to the local atmospheric moisture content.

Yet, moisture tracking models can only indicate where, under current conditions, evaporation is prone to precipitate. They do not capture atmospheric feedbacks resulting from added moisture by a TEAM system. Such feedbacks include the triggering of cloud formation by the lowering of the lifting condensation level (Figure 1) and potential changes of the local circulation. These feedbacks are expected to affect the formation and distribution of precipitation. Process based models including these feedbacks will be needed to further investigate the effects of TEAM on the evaporation footprints.

## Positioning of TEAM in the Field of Technologies for Freshwater Supply Enhancement

The development of TEAM for more precipitation is not in isolation. In this section, we position TEAM alongside (1) the most common method of freshwater production from seawater, reverse osmosis (RO), (2) another weather modification technology, cloud seeding, and (3) the nature-based approach of forestation for precipitation enhancement. The comparison is based on the main goal, suitable contexts, control and reliability, main costs, energy needs, and environmental risks of the technologies and is summarized in Table 1.

**Table 1:** Qualitative Comparison of Reverse Osmosis (RO), Cloud Seeding, Forestation for Rainfall Enhancement and TEAM on Several Characteristics of the Technologies

	TEAM	RO	Cloud seeding	Forestation
**Main goal**	Enhance downwind precipitation	Direct freshwater production	Enhance local precipitation	Enhance local or downwind precipitation
**Most suitable context**	Coastal regions where atmospheric moisture is too limited for precipitation and lifting mechanisms are present	Urban, arid coastal zones with infrastructure	Where cold clouds exist but lack ice nuclei	Moist regions with suitable land availability for afforestation
**Control & reliability**	Weather dependent and design dependent controllability	Mature, widely used, reliable technology for on-demand freshwater production	Widely used but low evidence base. Weather dependent	No control but for initial location selection. Weather dependent
**Main costs**	Spray: upfront investment and energy for deployment. Solar: upfront investment depending on material	Upfront investment and energy for deployment	Operational cost per deployment	Upfront investment, time investment
**Energy need**	Spray: pumping and spraying. Solar: limited, passive system	High: mainly pretreatment and pumping	Aircraft fuel	Low
**Environmental risks**	Weather modification risks and marine impacts. Spray: downwind salt aerosols	Brine discharge, marine impacts	Aircraft emissions, weather modification risks	Local drying, weather modification risks

Reverse osmosis (RO) uses high pressure at the saltwater side to force the water molecules to pass through a permeable membrane and produce freshwater.^36^ Its high controllability and reliability make it widely used in urban and industrial settings. While both RO and TEAM use seawater as input, TEAM’s goal is to enhance downwind precipitation, making it weather dependent and diffuse, and therefore better suited for rain-fed agriculture, ecosystem restoration, or climate adaptation. RO’s high operational energy demand for pretreatment and the RO process itself^37^ stands in contrast to TEAM. Spray technologies would need minimal pretreatment and substantially less energy for operation: Compare typical hydrostatic pressures of 55–80 bar for seawater RO^38^ versus creating a water spray at 100 m high which would need ∼11 bar (neglecting friction) from Bernoulli’s principle.^39^ Solar technologies operate passively, limiting energy consumption even further. However, for a full systems-level comparison, pumping and friction losses, energy loss by water droplets not fully evaporating for spray systems, and salt management in both RO and TEAM systems would need to be included, which may substantially increase operational energy needs beyond the idealized estimates given here. Capital costs of TEAM are expected to be largely determined by the design, size, the potential need to build offshore, and in the case of a solar technology the main material, while capital costs of RO are high, but known. Environmentally, brine discharge by RO installations can harm marine ecosystems,^40^ whereas TEAM’s distributed evaporation may mitigate brine accumulation, although marine impacts of offshore technology designs need to be assessed.

Cloud seeding introduces condensation or ice nuclei to existing clouds to enhance or sometimes reduce local precipitation.^9^ While cloud seeding is widely used, the evidence base for its effectiveness is low.^41–44^ However, there is evidence that glaciogenic seeding of cold-season orographic clouds can significantly enhance precipitation.^44^ Cloud seeding is constrained by cloud availability, and since it is mainly implemented through aircraft, expenses include fuel and environmental impacts are greenhouse gas emissions. TEAM differs in approach by acting at the start of the water cycle, not the end, by enhancing atmospheric moisture. It is therefore expected that TEAM could be more effective in enhancing convective precipitation in the warm season, in situations where atmospheric moisture, not nucleation, is the limiting factor in precipitation formation, and atmospheric lifting mechanisms are present. Both methods risk unintended weather changes, warranting precaution in research and implementation.^45^

Forestation is long known for its impacts on the water cycle. However, forestation with the goal of enhancing precipitation locally or downwind is an emerging idea in scientific literature.^3,10,46–50^ Forestation has clear cobenefits, such as carbon sequestration, biodiversity increase, and soil erosion control.^51,52^ It is similar to TEAM with a solar technology as both are large-scale, passive evaporation systems where control and reliability of the precipitation is low. Also, the energy need is low and the main costs are expected to be upfront investments. However, TEAM is expected to achieve higher water vapor fluxes since TEAM is not limited by biological water conservation strategies of vegetation. Furthermore, forestation depends on land availability and existing freshwater, and may risk local drying.^3,53^ TEAM on the other hand could be used in coastal regions unsuited for forestation, e.g because of high population density.

Overall, it will depend on the regional context, needs, and possibilities to decide which method is most suitable for freshwater supply enhancement, or if any other unconventional freshwater resource may be.^54^

## Outlook

In this perspective paper we have introduced the idea of Technology-Enhanced Atmospheric Moistening as a new method of large-scale freshwater generation via induced precipitation. We explained how and under which conditions TEAM could enhance precipitation using meteorological arguments, introduced technological options, and placed it in the context of the broader field of technologies for freshwater supply enhancement. We conclude this perspective with an outline of the research agenda to develop the idea further.

From the meteorological side, the main question that needs to be answered is can moistening the atmosphere increase precipitation in a certain region, and if so, how much moistening is needed to increase precipitation significantly? Using a moisture connections data set, we could make an initial estimate of where extra evaporation could lead to enhanced precipitation. However, TEAM will impact the atmospheric processes that lead to precipitation. This highlights the need for the use of tools to quantify the relationship between TEAM and regional precipitation. These tools can range from simple process based conceptual models, e.g. the convective boundary layer slab model,^55–57^ to numerical models such as Large Eddy Simulation^58,59^ and numerical weather prediction models.^60–62^

From the technological side, the main question that needs to be answered is how can we provide the needed amount of atmospheric moisture to enhance precipitation? Laboratory experiments are the first step in answering this question. However, feedbacks between the technologies and the atmosphere will control the effective moistening impact that a certain technology can have. Results from laboratory and field experiments will in turn inform meteorological research and vice versa in an iterative manner. Incorporating TEAM technologies into atmospheric models can also support the design of these systems. For example, modeling can help determine the optimal height of a spraying technology or the required surface area of a solar evaporation technology to supply sufficient moisture for enhancing precipitation under specific atmospheric conditions.

When TEAM research and development has advanced, more disciplines should be involved to investigate the regional effects of TEAM implementation on hydrology, ecology, and society. We believe that collaboration between all these disciplines is essential for the advancement of TEAM and should form an integral part of future regional design for TEAM implementation. Within such regional design, three crucial topics for further research can be identified: the fate of the precipitation, the ecological impacts, and the socio-economic costs and benefits, which we will shortly discuss here.

A key question for regional design is what happens to TEAM-induced precipitation once it reaches the surface. Its fate will determine whether and how it can contribute to freshwater availability. Depending on local climate, topography, soil type, and land use, precipitation may infiltrate into the soil, directly benefiting local ecosystems or agriculture and recharging aquifers, or it may runoff into rivers or reservoirs. Hydrological studies are needed for the quantification of this regional moisture partitioning. Harvesting methods could be included in the design, such as collecting runoff or water from roofs and streets and storing it in the soil or in tanks,^63^ which could increase the value of the precipitation for e.g. agriculture.

Besides the hydrological impact, TEAM implementation will impact ecology. First, there are the impacts of offshore structures and potentially enhanced salinity on marine ecosystems to be considered and limited as much as possible. Second, TEAM may affect terrestrial ecosystems both directly through changes in atmospheric humidity and temperature, and indirectly through changes in soil moisture availability and variability.^64^ These changes may be considered positive or negative, depending on the goal of the implementation. It is important that ecological impact assessments are made as part of the regional design process.^65^ When the goal of implementation is ecosystem restoration, the amount of precipitation that is needed for a certain ecosystem needs to be estimated.

Lastly, regional design should asses the socio-economic costs and benefits of implementing TEAM compared to other methods of freshwater production, while considering potential cobenefits of enhanced precipitation such as counteracting salt accumulation in soils^4^ or improving air quality in polluted coastal regions through enhanced wet deposition of particulate matter.^66^ Since weather modification through TEAM partly fits into the United Kingdom Royal Society’s definition of geoengineering,^67^ ethical frameworks for geoengineering^68–72^ can be used as guidelines for responsible research and implementation.

With continued research and technological innovation tailored to TEAM for more precipitation, this approach has the potential to become a solution for large-scale freshwater needs.
